# New Food Crop Domestication in the Age of Gene Editing: Genetic, Agronomic and Cultural Change Remain Co-evolutionarily Entangled

**DOI:** 10.3389/fpls.2020.00789

**Published:** 2020-06-11

**Authors:** David L. Van Tassel, Omar Tesdell, Brandon Schlautman, Matthew J. Rubin, Lee R. DeHaan, Timothy E. Crews, Aubrey Streit Krug

**Affiliations:** ^1^The Land Institute, Salina, KS, United States; ^2^Department of Geography, Birzeit University, Birzeit, Palestine; ^3^Donald Danforth Plant Science Center, St. Louis, MO, United States

**Keywords:** domestication, coevolution, gene editing, agronomy, cultural evolution, new crops

## Abstract

The classic domestication scenario for grains and fruits has been portrayed as the lucky fixation of major-effect “domestication genes.” Characterization of these genes plus recent improvements in generating novel alleles (e.g., by gene editing) have created great interest in *de novo* domestication of new crops from wild species. While new gene editing technologies may accelerate some genetic aspects of domestication, we caution that *de novo* domestication should be understood as an iterative process rather than a singular event. Changes in human social preferences and relationships and ongoing agronomic innovation, along with broad genetic changes, may be foundational. Allele frequency changes at many loci controlling quantitative traits not normally included in the domestication syndrome may be required to achieve sufficient yield, quality, defense, and broad adaptation. The environments, practices and tools developed and maintained by farmers and researchers over generations contribute to crop yield and success, yet those may not be appropriate for new crops without a history of agronomy. New crops must compete with crops that benefit from long-standing participation in human cultural evolution; adoption of new crops may require accelerating the evolution of new crops’ culinary and cultural significance, the emergence of markets and trade, and the formation and support of agricultural and scholarly institutions. We provide a practical framework that highlights and integrates these genetic, agronomic, and cultural drivers of change to conceptualize *de novo* domestication for communities of new crop domesticators, growers and consumers. Major gene-focused domestication may be valuable in creating allele variants that are critical to domestication but will not alone result in widespread and ongoing cultivation of new crops. Gene editing does not bypass or diminish the need for classical breeding, ethnobotanical and horticultural knowledge, local agronomy and crop protection research and extension, farmer participation, and social and cultural research and outreach. To realize the ecological and social benefits that a new era of *de novo* domestication could offer, we call on funding agencies, proposal reviewers and authors, and research communities to value and support these disciplines and approaches as essential to the success of the breakthroughs that are expected from gene editing techniques.

## Introduction

*De novo* domestication of new crops from currently wild plants could help solve a wide range of problems, including genetic and species diversification of agricultural systems (Fernie and Yan, 2019); expansion of agricultural production onto degraded sites, stressful environments, or regions highly vulnerable to climatic extremes (Zhang et al., 2018); improvement of the fit between crops and particular local ecological niches (Fernie and Yan, 2019); and intensification of the range of vital ecosystem services provided by crops (Weißhuhn et al., 2017). Many new food crops will need to produce larger, more harvestable tubers, roots, fruits and seeds. Because this kind of change is both visually obvious, including in archeological records, and something that evolved independently many times in the past, it is not surprising that these are the core traits of the domestication syndrome for food plants (Doebley et al., 2006; Dong et al., 2019; Woodhouse and Hufford, 2019) and attractive targets for genetic modification.

In much of the recent *de novo* food crop domestication literature, domestication and the fixation of alleles conferring the domestication syndrome are used as interchangeable concepts (discussed below). However, our definition is closer to that of Harlan (1992), who describes broader changes: “Domesticated plants are those brought into the *domus* [Latin for household] which may mean the dooryard, garden, field, orchard, vineyard, pasture or ranch. It may also include yards, parks, cemeteries, golf courses, roadsides, forests, and other managed areas. In ecological terms it is the change in habitat that is critical…. domestication tends in the direction of making the plant populations dependent on human interference and man-made habitats. Since the processes of domestication are evolutionary in nature, all intermediate degrees and conditions may be expected, but a fully domesticated plant is entirely dependent on human intervention for survival.”

Advances in gene editing technological interventions, such as CRISPR/Cas9, could enable a new era of *de novo* domestication (Østerberg et al., 2017; Chen et al., 2019; Eshed and Lippman, 2019; Fernie and Yan, 2019; Khan et al., 2019; Wolter et al., 2019) defined as “the introduction of domestication genes into non-domesticated plants” (Fernie and Yan, 2019). Several labs have independently and successfully modified domestication-related genes in wild species as proof-of-concept (Lemmon et al., 2018; Li et al., 2018; Zsögön et al., 2018). Discovering or creating favorable alleles of relatively simply inherited “domestication genes” (DGs) (Doebley et al., 2006; Østerberg et al., 2017; Khan et al., 2019) – whether through classic selective breeding, mutagenesis or genome-editing – is helpful during *de novo* domestication.

Such research could contribute new understanding and opportunities, particularly in well-characterized genes such as the seed dormancy gene G, which appears to have been selected in parallel during the domestication of several crops in at least three plant families (Rendón-Anaya and Herrera-Estrella, 2018). Gene editing and similar technologies may be necessary to produce effective DG alleles on a realistic timeframe for the *de novo* domestication of some wild plant taxa (Eshed and Lippman, 2019; DeHaan et al., 2020) and some authors recognize that novel variation must then be introduced into diverse germplasm (Lemmon et al., 2018) and “tuned” by traditional selection on standing variation of many small-effect loci (Eshed and Lippman, 2019).

However, other recent gene editing papers may inadvertently oversimplify the rich, complex process of plant domestication. Some imply that prehistoric domestication happened when “simple choices ultimately led to the pyramiding of valuable mutations and re-combinants in key genes” (Fernie and Yan, 2019) and emphasize the importance of monogenic domestication traits (Li et al., 2018). Zsögön et al. (2018) state that the editing of six genes demonstrates “that targeted reverse genetic engineering of wild plants could rapidly create new crops.”

The availability of new tools such as CRISPR, and the possibility of applying them toward *de novo* domestication of novel crops, could be misunderstood by society to mean that gene editing can produce domestication as a nearly instant and/or inexpensive event, failing to recognize the many years of foundational research that was required to characterize and identify candidate genes and develop transformation and tissue culture regeneration systems (Van Eck, 2018). The tendency to emphasize the speed and ease of domestication of plants such as *Solanum pimpinellifolium* (currant tomato) via gene editing is illustrated in the following headlines and statements: “CRISPR can speed up nature… It took thousands of years for humans to breed a pea-sized fruit into a beautiful beefsteak tomato. Now, with gene editing, scientists can change everything” (Hall, 2018); “Gene editing can potentially cram millennia of agricultural progress into the blink of an eye” (Keats, 2019); “Plant breeding at the speed of light: The power of CRISPR/Cas…” (Wolter et al., 2019); “CRISPR/Cas brings plant biology and breeding into the fast lane” (Schindele et al., 2020); “The CRISPR technique quickly tamed ‘unruly’ ground cherries” (Saey, 2018); “For the first time, researchers have created, within a single generation, a new crop from a wild plant…by using a modern process of genome editing” (University of Munster, 2018); “Tweaking just a few genes in wild plants can create new food crops” (Hereward and Curtis, 2018).

While new gene editing technologies may accelerate some genetic aspects of domestication, we caution that *de novo* domestication resulting in widespread and ongoing cultivation should be more accurately understood as an ongoing, iterative process rather than a singular event. New genetic techniques may allow us the opportunity to begin – not to bypass – this “lengthy and tedious” (Wolter et al., 2019) work. Whereas earlier researchers emphasized the ways that humans had discovered loss-of-function plant mutations during domestication, leaving plants dependent upon farmers for their defense and dispersal (Zohary and Hopf, 2000; Anderson, 2005), the emerging consensus is that domestication is a long-term, co-evolutionary deepening of a mutualistic relationship, involving cultural, technological, biological, and ecological factors (Zeder et al., 2006; Gepts, 2010; Meyer et al., 2012; Meyer and Purugganan, 2013; Gremillion et al., 2014; Larson et al., 2014; Allaby et al., 2017; Swanson et al., 2018; and see especially the review by Zeder, 2015).

Historically, the genetic changes that have come to distinguish domesticated plants from their wild relatives are the outcome of an interaction of sociocultural and environmental pressures reflecting broader changes in human preference and agronomic intervention. In domestication, culturally innovative changes in human behavior that result in environmental modification (such as preparing soil and plant harvesting and processing) are “entangled” with biologically innovative changes in plant genetics (Fuller et al., 2010). Domestication is an ongoing process since people and plants live in dynamic social-ecological systems.

**FIGURE 1:**
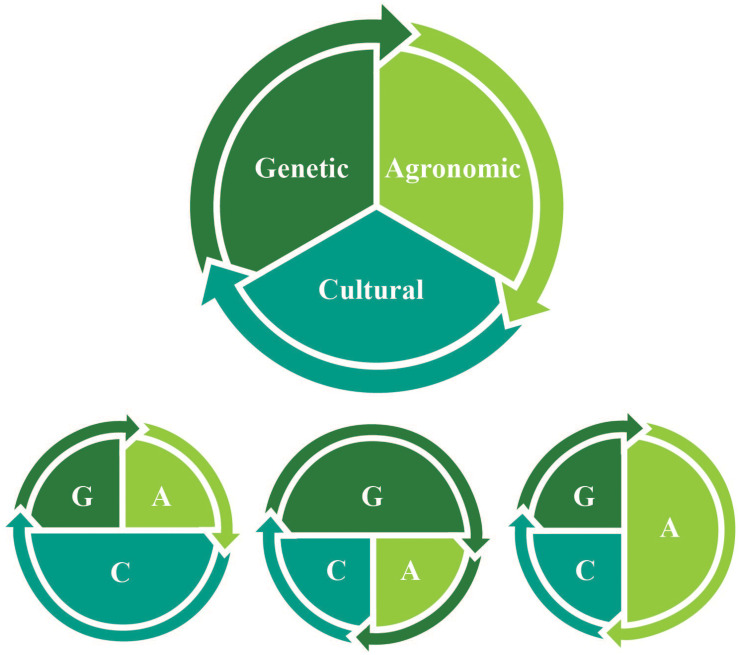
Varying magnitudes of genetic (G), agronomic (A), and cultural (C) change and their interactions drive *de novo* domestication processes.

**FIGURE 2:**
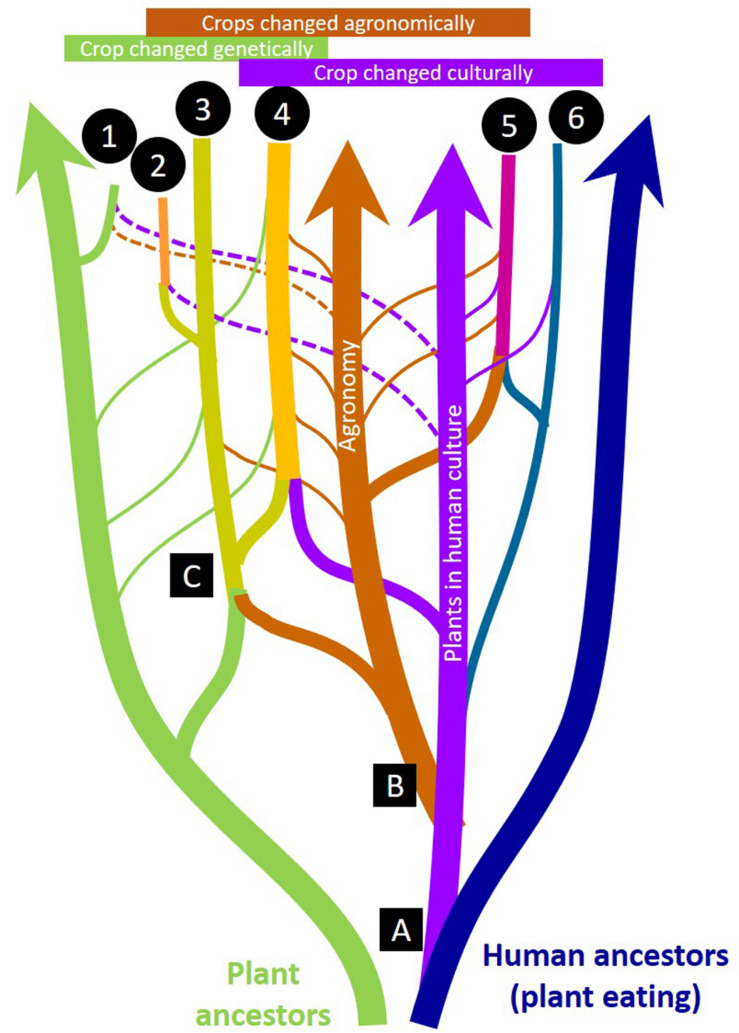
Genetic, agronomic, and cultural changes are co-evolutionarily entangled in crop domestication. A reticulate biological/cultural “evolutionary tree” is shown in cartoon form (not to scale) to illustrate how “lineages” of plants, agronomic technology/traditions, and inherited human ethnobotanical culture “hybridize” to create different kinds of crops: (1) *De novo* incipient new crops from wild plants by breeding or gene editing (e.g., silphium), (2) New food crops developed from non-food crops by breeding or gene editing (e.g., intermediate wheatgrass), (3) Forage, fiber, and energy crops (e.g., alfalfa), (4) Major world food crops (e.g., maize, rice), (5) Cultivated crops with little genetic change (e.g., cranberry), (6) Culturally important wild-crafted food and medicinal plants (e.g., ‘akkoub). Major evolutionary innovations are numbered: (1) Cultural references to plants (names, stories, recipes, etc.) appear, (2) Agronomic practices appear, (3) Genetically distinct cultigens appear. Narrow lines show “horizontal” influences such as accidental or deliberate gene flow between cultigens and wild relatives (narrow green lines) or changing cultural uses of harvested plants (narrow purple lines) or the influence of new agronomic practices and technologies (narrow brown lines). Dotted narrow lines indicate a very recent or emerging influence.

A more interdisciplinary approach is therefore needed to inform the review and implementation of new gene editing research on *de novo* domestication. We provide a practical framework that highlights and integrates multiple drivers of change (Figure 1) to conceptualize *de novo* domestication for communities of new crop domesticators, growers and consumers. Various magnitudes of genetic, agronomic, and cultural change drive cycles of selective pressures that result in the fixation of domestication genes – and broad genetic changes, innovations in management, and social support may all be required to enable domestication processes to continue at different pivotal moments and for the long term across human generations and geographies (Table 1). To realize the ecological and social benefits that a new era of *de novo* food crop domestication could offer, it is necessary to recognize how genetic, agronomic, and cultural changes remain co-evolutionarily entangled (Figure 2).

**TABLE 1:** Common evidence, processes, and participants and practices involved in the genetic, agronomic, and cultural drivers of *de novo* domestication and crop improvement.

**Evidence: sources and results of domestication**	**Processes: iterative cycles of transmission and change in domestication**	**Participants and practices: major stakeholders and methods to accomplish domestication and crop improvement**
**Genetic**		
• Extant and new cultigens • Archeological remains of extinct cultigens that are morphologically distinct from wild ancestors	• Plant lifecycle from seed to seed (months to a few years) • Selection of new scions to use in grafting (decades) • Division of tubers, rhizomes (yearly) • Formal breeding cycles, gene discovery projects (months to a few years)	• Germplasm collections at gene banks, botanical gardens • Traditional landrace development by farmers through visual or unconscious selection for reduced shattering, seed dormancy • Public and private plant breeding using sexual recombination and selection • Public or private genetic engineering (cisgenic, transgenic) • Seed exchange networks and community gene banks
**Agronomic**		
• Horticultural practices and skills • Management knowledge • Tools and technologies	• Training of children by families and communities (decades) • Formal educational degrees and certifications (years to decades) • Publication of major new books, articles (years to decades) • County fairs (yearly) • Farmer field days, conventions (yearly)	• University/government agronomy research into best practices for germination, inoculation, soil fertility, maintenance, harvest, storage, etc. • Journalistic and ethnobotanical interviews, publications • Development of integrated pest control strategies • Engineering of specialized machinery for harvesting or other agronomic activities • Formal and informal centers for agricultural education and extension • Practitioner innovation associations
**Cultural**		
• Laws and policies • Educational practices, both formal and informal • Stories, recipes, and artworks • Values, attitudes, beliefs, and norms	• Election cycles (years) • New companies or corporate leadership (years to decades) • Social movements (years to decades)	• NGOs hosting citizen science projects • National and provincial government dietary recommendations, public health campaigns • Cultural production and circulation through art, literature, cookbooks, digital and social media • Commercial and NGO advocacy, lobbying, and marketing communications • Commodity associations funding research and marketing • Regional cuisine change through immigration, cultural diffusion, urbanization, and travel • Political parties propose policy and spending, seeking votes, contributions from special interests

## Genetic Change Beyond Domestication Genes

Wild plants can be regarded as reservoirs of useful genes (Zhang et al., 2018) needed to create “climate-smart crops” (Li et al., 2018). However, the view that this genetic resource can be tapped by cleanly switching out a few domestication genes, hopefully “without causing an associated drag on other useful traits” (Li et al., 2018) is overly optimistic because it underestimates the additional genetic changes that will be required to improve wild plants’ ability to respond to favorable environments, high plant density, increased population, and human care. Domestication is not a single genetic transformation. Other kinds of plant genetic research and genetic change are needed beyond and/or preceding modification of DGs.

### Identification and Preparation of Candidates

Research may be needed to identify potential candidates for domestication (Ciotir et al., 2019), to rank species or subspecies by predicted breedability (e.g., genome size, ploidy, mating system), to identify barriers to human use (e.g., toxicity), and to prepare wild germplasm for gene-editing (e.g., genomic sequencing, mapping) (DeHaan et al., 2016). The foundation of a domestication program includes obtaining and characterizing the wild germplasm (Schlautman et al., 2020) and ascertaining that additive genetic variation for crop yield and disease resistance are available in the gene pool. A successful domestication candidate should come from a sufficiently large primary or secondary gene pool to ensure sustained breeding, as domestication by any means is only the beginning of continuous cycles of breeding for yield, quality and resistance.

### Core Germplasm Development and “Tuning” of DGs

Domestication via gene-editing almost certainly implies a severe genetic bottleneck because it currently requires an efficient plant transformation/regeneration protocol (Chen et al., 2019; Fernie and Yan, 2019). Tissue culture protocols are not available for most wild plants or every genotype of crop plants; even where many amenable genotypes are available, genetic modification is laborious and may be constrained by intellectual property protection issues, thereby limiting the number of transformed lines that will be rapidly available to farmers and breeders. Mutational load could increase by drift during such a bottleneck (or later through hitchhiking with DGs) as appears to have happened during maize domestication (Wang et al., 2017). Pre-domestication inbreeding could be attempted to estimate mutation load and thus identify candidates with lower levels of preexisting load, or even to begin to purge it. Alternatively, edited genes could be immediately introgressed into pre-identified genetic diversity panels to reverse the bottleneck effect (Eshed and Lippman, 2019; Fernie and Yan, 2019), although recovering homozygosity for the edited allele while avoiding genetic bottlenecking will require sophisticated crossing strategies. Reversing bottlenecks, identifying unpredicted genetic background effects (Wolter et al., 2019), and the general need to “tune” the expression of novel major-effect alleles (Eshed and Lippman, 2019) all argue that extensive post-domestication breeding should be expected in addition to pre-domestication work.

### Breeding for Vigor and Broad Adaptation

All the world’s major crops are, almost by definition, grown far beyond the environment of their origin. They have adapted to many soil types, climates and daylengths. To provide a positive return on the research investment and to attract sufficient cultural notice as to become part of human cuisine, *de novo* domesticates will arguably need to be broadly adapted. Candidate species should be evaluated in multiple environments to identify those capable of broad adaptation or those with genetic variation for yield stability in multiple years and environments. Evaluation of breeding lines in multiple environments permitted the development of broadly adapted wheat varieties. Norman Borlaug advanced only varieties “that withstood the rigors of both environments,” and considered broad adaptation to have been one of the three greatest successes of wheat breeding in the late twentieth-century (Borlaug, 1983). With increasing climate instability, new crops must be able to withstand different rigors even in a single location, within or across years.

### Breeding for Biotic and Abiotic Stress Tolerance

Winter hardiness (for perennials and winter annuals) and disease/insect resistance are specific adaptation traits that DG editing will not improve for undomesticated plants. Editing/engineering of resistance (R) genes could help, of course, although R genes are more likely to be species-specific “orphan” genes than DGs (Woodhouse and Hufford, 2019) and therefore researchers may rarely be able to take advantage of the gene discovery work already accomplished for existing crops. Crop pathogens reduce harvests by about 20% globally (Bebber and Gurr, 2015). Wild plants are not immune to pathogens; fungicide application increased plant biomass by 31% in a native North American grassland (Mitchell, 2003). The cultivation of wild plants could create conditions that favor the spread of some of their pests and pathogens, for example, by increasing the number, density, size and uniformity of host plants (Chen et al., 2018). Breeding for increased biotic stress resistance may therefore be necessary for new crop domestication and plant pathologists recommend that breeders include selection for quantitative and broad-spectrum resistance traits (Bebber and Gurr, 2015) in addition to R gene mediated resistance.

### Breeding for Yield and Quality

Traits controlled by many genes are poor candidates for genome editing and improvement may require more conventional cycles of breeding for the foreseeable future. While some important agronomic traits are monogenic, domestication traits in some crops are polygenic (Hämälä et al., 2019). Flavor, ripening, post-harvest physiology, and nutritional profile are important traits for food crops and unlikely to be completely satisfactory in “crops” undifferentiated from wild ancestors except at a few DG loci and are likely to be genetically complex traits. For example, Zhang et al. (2015) found 28 volatile chemicals to be involved in tomato flavor/aroma and associated with 125 markers. Seed set (or seed fertility), the percentage of florets producing a viable seed, has been found to be quite low in some wild plants both in their wild habitats and under cultivation (Cornelius, 1950). However, recurrent phenotypic selection can improve this trait (Marshall and Wilkins, 2003), which would improve the yield and breedability of crops developed by gene editing. The conventional modeling of yield as a genetically complex trait with many small-effect loci continues to be supported (e.g., Martínez et al., 2016). Evidence that genetic correlations between agronomic traits constrained and slowed maize domestication and that genetic constraints increased during domestication (Yang et al., 2019) suggests that – for some species at least – it will be hard to identify many agronomic targets for genetic modification that have large, positive, and independent effects.

### Value of Recognizing Broad Genetic Changes Involved in Domestication

The previous section shows that recognizing genetic change in the broader context of ongoing domestication can lead DG-based project teams to invest appropriate time, resources, and strategic planning to domestication efforts in the form of careful wild candidate selection and screening, collection, characterization and pre-adaptation of germplasm. Funding agencies should recognize the need to support multi-location, multi-trait breeding of new domesticates even after successful DG improvement.

One practical recommendation for accelerating *de novo* domestication of food crops and bypassing some stages described above is to focus on species that have been domesticated as forage, ornamental, medicinal, bioenergy or timber crops. These are species that have responded to agricultural conditions and human management and may have been further selected by breeders for adaptation to managed environments and tolerance of multiple soils and climates. Specific agronomic recommendations and laboratory protocols may be available. Last, but not least, the plant may already be culturally familiar to farmers and policy makers. Presumably, if it has become a successful forage or other kind of crop, this species has been found by humans to be pleasant to work with, non-invasive, and aesthetically compatible with rural landscapes. Forage crops do not require most of the traits conferred by DGs, yet traditional breeding has improved forage yield and usefulness to farmers.

In contrast, even locally adapted native species have been difficult to use as planted forages without some domestication (Morrison, 2016). Despite a wealth of native grass species, not a single native species is used widely enough to merit keeping statistics on the acreage of seed production in the United States (National Agricultural Statistics Service, 2019). Interestingly, the National Agricultural Statistics service primarily differentiates hay acreage as “tame,” “wild,” or “alfalfa” (National Agricultural Statistics Service, 2019), supporting our view that few forage species have achieved deep cultural value (Figure 2). Poor seedling establishment and vigor is an example of a trait that constrains adoption of forages (Vogel, 2000). DGs leading to increased seed size could improve these traits but about half of the variation in seedling vigor is unrelated to seed size (Vogel, 2000). The value of recurrent selection for the performance of both native and introduced forages illustrates the fact that the micro-environments created by the human-crop mutualism are novel (DeHaan et al., 2007; Purugganan, 2019).

*Thinopyrum intermedium* (Host) Barkworth & D.R. Dewey (intermediate wheatgrass, hereafter) is being developed as a new perennial cereal grain marketed under the trade name Kernza (DeHaan and Ismail, 2017). This new crop is an example of a candidate for DG editing (DeHaan et al., 2020) that builds upon an established forage with extensive germplasm enhancement and multitrait breeding. Intermediate wheatgrass was collected from the wild in the former USSR in the 1930s (Hanson, 1959). Forage varieties were developed in the United States and Canada from 1945 onward by selection from the wild germplasm for vigor and seed fertility (Vogel and Hendrickson, 2019). Recurrent mass selection with controlled interpollination of selected genotypes was used to increase seed yield in the 1950s and other programs emphasized lodging resistance, plant health, productivity, forage quality and broad adaptation (Vogel et al., 1993). By 1988, 500 tons per year of seed was being produced for sale in Saskatchewan alone (Saskatchewan forage seed development commission, 1998). In response to the suggestion that new perennial grains could conserve soil, the Rodale Institute began evaluating wild perennial grasses as candidates in 1983 (Wagoner and Schaeffer, 1990; Wagoner and Schauer, 1990). After scoring ten agronomic traits, including seed size, seed shattering, and stem lodging, intermediate wheatgrass was identified as the top candidate and two cycles of recurrent selection for improved yield and harvestability were performed at the USDA-NRCS Big Flat Plant Materials Center (Cox et al., 2002). Forage varieties were included along with wild accessions in the starting population from which the Rodale Institute began its selections (Wagoner and Schauer, 1990). In the early 2000s, The Land Institute revived the intermediate wheatgrass domestication program and has since performed nine cycles of recurrent selection; three cycles have been completed at the University of Minnesota (Crain et al., 2020). Recent cycles of selection use genomic selection to accelerate genetic gains for domestication traits and cereal grain yield (Zhang et al., 2016).

Intermediate wheatgrass is a challenging species for genome editing. No transformation protocol is available, the species is highly heterozogous, the genome contains more than 11 million base pairs, and the genome is a complex allohexaploid (DeHaan et al., 2020). Domestication phenotypes due to knockouts in this species may require obtaining plants with all six alleles in the edited non-functional form. Such an effort involves not only completing gene edits but also possibly breeding them to homozygosity. Clearly, intermediate wheatgrass would not be a candidate for DG editing if the crop had not previously undergone more than three decades of breeding and agronomic work with the objective of obtaining a successful perennial grain crop. In essence, DG editing of this species has potential to dramatically improve its functionality as a grain crop by providing breakthrough improvements in one or two domestication traits because the species has already been the target of numerous breeding, agronomy, and utilization efforts (Tyl et al., 2020).

Genetic studies with intermediate wheatgrass thus far have revealed both the activity of known domestication genes from other grain crops in this new species, but also the potential challenges to utilizing such genes. Larson et al. (2019) identified 42 candidate genes in intermediate wheatgrass that can influence traits relevant to domestication. These genes are potential targets for selection or editing. However, even for the traits with highest heritability, such as free threshing ability, 16 significant markers associated with the trait were detected. This indicates that a large number of genomic regions were found to influence the trait, rather than a single high-impact locus. Furthermore, the full marker set only explained about 25% of variation in some environments, and marker effects depended upon environment in many cases. Therefore, even for simple domestication traits, there can be substantial interaction between genes and environment, and influence of a particular allele may depend on genetic “background,” or interaction with the rest of the genome. These realities could greatly increase the complexity of genome editing approaches; perhaps particular allele forms will be necessary to override gene by environment interaction, or careful breeding will be necessary to preserve a complementary background genome that will not override the novel domestication allele introduced through editing.

## Agronomic Change

The increased value of a domesticated plant to humans, compared with its wild ancestor, depends on its altered phenotype (e.g., large seeds, high seed yield, and reduced branching). Variation in plant phenotypes result from both differences in environment (E), genes (G) and the interaction of G × E. From the perspective of growers, the exact contribution of G or E or G × E is irrelevant; the final phenotype is relevant. In addition to G × E, agronomic management (M) changes highly relevant aspects of the environment (Hatfield and Walthall, 2015; Wang et al., 2019). There is variation in management and this variation can be transmitted across human generations, constituting a necessary component of domestication processes.

Agronomic change occurs when farmers learn to produce specific abiotic and biotic environmental changes that enhance plant productivity and harvestability (Altieri, 2004). Some of these changes are more-or-less permanent, whereas new seed must be planted each year. Perhaps this is one reason why we more often think about the genetic than the agronomic contributions to domestication. We can grow crops side by side with ancestors whereas our ancestors modified vast landscapes to increase and stabilize crop yield. They drained wetlands, terraced mountains, cleared forests, and killed off large wild herbivores and it is difficult to experimentally replicate these changes at research stations.

Genetic change in plant domestication is interdependent with the actions taken by early farmers (intentionally or unintentionally) to prepare an environment that was consistently favorable for plants they preferred (Doebley et al., 2006; Clement et al., 2015; Altman and Mesoudi, 2019; Edwards et al., 2019; Mueller, 2019). The iterative development of plants makes their final size, shape and fecundity extremely plastic compared with animals. The influence of environment upon the realized yield of all crops – regardless of their yield potential in optimal environments – is so intuitively understood by any gardener or farmer, so central to the purpose of entire agronomy departments, and so inherently complicating to plant breeding that perhaps it is easy to overlook when considering *de novo* domestication. As an example, weed control alone doubles soybean and corn yields in the corn/soy region of North America (Soltani et al., 2016, 2017) and this estimate was made for crops otherwise managed according to modern best-practices. Without high quality seed, uniform seed placement and row spacing, well-managed fertility, etc., weeds would likely have been more competitive.

Agronomic change encompasses a wide range of ecosystem alterations, from the straightforward replacement of native vegetation with crop species, to dramatic interventions in a wide range of factors that affect crop productivity, including water, temperature, nutrient, weed, insect, and pathogen management. Before the fossil fuel era, agronomic changes were primarily “ecological” in that farmers manipulated key ecosystem patterns and processes to favor productivity per unit human or animal labor (Smil, 2018). Examples of such practices included the use of crop rotations, the integration of legumes, use of periodic flooding, and as mentioned, elimination of competing vegetation with weeding (Gliessman, 2015). The discovery and rapid increase in availability of fossil fuels profoundly relaxed the energetic constraints on possible agronomic practices. People have successfully employed fossil-fuel based strategies to relax or eliminate almost every limiting factor to crop productivity, including synthetic nitrogen, biocides, plastic row covers, pumping and transportation of water for irrigation, and more (Pimentel and Pimentel, 2007). As we once again move toward cultural expectations of reduced fossil fuel dependence, we see agronomic practices shifting as well from approaches that maximize fossil fuel-dependent agronomic practices in order to maximize yields, toward agronomic solutions that maximize ecological intensification in order to optimize yields (Crews et al., 2016).

### Value of Recognizing Agronomic Change Involved in Domestication

Wild species being introduced into an agricultural environment are likely to exhibit dramatic plasticity as they are released from some forms of herbivory and competition from other plant species. However, not all species or genotypes are equally plastic; some may require additional environmental modification to thrive. Since farmers are in the business of modifying the environment to benefit plants, it makes sense to screen *de novo* domestication candidates in conditions characteristic of the target agricultural system, whether that be an irrigated paddy or an orchard, a high-input cash plantation or a low-input farm. Plants without the plasticity to adapt to the target environment are poor candidates for domestication.

Learning the ideal conditions for each candidate will require either rigorous agronomy or trial-and-error experimentation by farmers. Time and funding to permit adequate characterization of crop candidate response to variations in soil texture, fertility, water availability, cold stratification and vernalization as well as other ecological factors should be included in any *de novo* domestication project.

For wild plants never previously used as crops, the research investment in agronomic drivers of domestication may be substantial and time-consuming – just as with the research investment into genetic change. Recognizing this may prompt *de novo* domestication research teams to attend to agronomic change and introduce a completely wild plant species as a crop for forage, erosion-control, pollinator habitat, or niche high-value use of seeds or fruits (medicinal, specialty vegetable or flavoring, cosmetics) prior to investing in genetic changes necessary for a major food crop.

Intermediate wheatgrass domesticators in the 1980s benefitted from planting practices, weed control, and fertilization that are recommended to farmers and researchers (Wills et al., 1998). Some of these recommendations may turn out to be counterproductive for seed producers vs. their intended audience of forage producers, but valuable insights for driving domestication via managing a new perennial cereal crop were available from forage grass seed producers (Horton et al., 1990; Saskatchewan forage seed development commission, 1998; Hybner and Jacobs, 2012; Kruger, 2015).

## Cultural Change

From an evolutionary perspective, domestication is an example of co-evolution between mutualists. Genetic changes in human partners during this co-evolutionary process are modest, though documented (Altman and Mesoudi, 2019). However, human culture can evolve by accumulating complex behaviors and transmitting them to successive generations (Altman and Mesoudi, 2019). Some of these behaviors relate to the knowledge, practices and tools used in tending crops, i.e., resulting in agronomic change as described above.

Beyond applied horticultural tools and knowledge, other cultural changes are required to enable and sustain a deepening, long-term human relationship with a new crop. Past human cultural evolution enabled initial domestication and agricultural niche construction (see review in Laland (2017). Now, cultural change describes the human community valuation of a species in social, economic, and culinary terms that may be necessary for *de novo* domestication processes to begin, continue, and succeed in bringing a new crop into widespread cultivation and use.

Human management of plants includes a variety of activities – gathering, tolerating, enhancing, and protecting *in situ* as well as *ex situ* sowing and transplanting – which suggests multiple possible routes for domestication processes (Casas et al., 1996; Lins Neto et al., 2014). Sociocultural factors shape these human choices about management and thus, for example, which plant species are engaged in incipient landscape domestication (Betancurt et al., 2017). Diverse cultural knowledge and methods of enculturation also shape how innovation, including domestication processes and products, are pursued and maintained across generations (Larson et al., 2014) with both negative and positive social and ecological implications (Scott, 2017; Swanson et al., 2018), such as the development of cooperative behavioral norms (Zeder, 2016).

The ongoing co-evolution that defines the domestication relationship must be sustained, on the human side, by society. Some degree of cultural change in response to a potential new crop, such as at the community scale, may be required in order to influence human resource allocation (Zeder, 2015) and successfully build investments to pursue agronomic and/or genetic change in terms of breeding and management experimentation, training of farmers, and the development of food processing, storage and distribution techniques.

*Vaccinium macrocarpum* (cranberry, hereafter) is an example of successful, ongoing crop domestication accomplished primarily through human cultural and agronomic change with minimal plant genetic change so far. A native North American fruit crop long wild-harvested by indigenous peoples and then adopted by European settler colonists, cranberries were valued as winter sources of calories and nutrients, especially vitamin C. They were so important that laws were passed banning the collection of the wild fruits prior to a certain date each fall (Klingbeil and Rawson, 1975). Though formal documentation is lacking, we consider it likely that indigenous peoples managed wild cranberry stands for increased yield (Doolittle and Mabry, 2006).

Agronomic change in cranberry domestication was advanced when transplanting of wild vines began in 1816 (Eck, 1990) and as cultivation spread with increasing economic returns by mid-century (Peltier, 1970). Over the next 100 years, the “big four” cultivars of selected clones from wild genotypes were involved in increasing agronomic and cultural change, including: managing cranberry hydrology using dikes and canals, plant propagation through sanding, protection from biotic (insects) and abiotic pressures (cold) by flooding, and harvest using specially designed cranberry rakes, barrels, crates, and eventually machines (Cole and Gifford, 2009); and organizing into cooperatives (i.e., Ocean Spray), who in turn developed new cranberry products (juices, sauces), creating cranberry growers’ associations, establishing cranberry experimental stations, planning festivals to celebrate cranberry flowering and harvest, and establishing traditions like crowning an annual cranberry queen (Peltier, 1970; Eck, 1990; Schlautman, 2016).

Nearly all the agronomic and most of the cultural change widely recognized and practiced were accomplished by the time genetic change (artificial selection) produced any sort of germplasm that was adopted by cranberry growers. The first cranberry breeding program was initiated in 1929 after growers and scientists had been unable to address the problem of cranberry false blossom disease through agronomic domestication (Eck, 1990). In 1950 the first cranberry cultivars were released after one cycle of selection from the 1929 program. A few of these varieties were eventually adopted because of their increased productivity and their perceived improved yield stability, especially cultivar “Stevens.” Most cranberry breeding programs were abandoned shortly after the 1950 releases. Rutgers University started a program in the late 1980s, the University of Wisconsin started a small program in the 1990s, and a Wisconsin grower independently started a private breeding program in the 1990s (Schlautman, 2016). The next set of varieties with improved productivity were released beginning in the early 2000s (Fajardo et al., 2013), and those varieties are being adopted and planted (Gallardo et al., 2018). However, cranberry growers still plant older varieties and even continue to plant and harvest beds of cranberry clones selected from the wild in the early 1800s (Vorsa and Zalapa, 2019).

DG traits have yet to be identified in cranberry. A recent survey revealed that cranberry growers perceived fruit quality traits and, specifically, fruit firmness as needing the most attention from breeders. This is likely in direct response to premiums being paid by cranberry processors for fruits that meet standards for sweetened dried cranberry processing, while prices for cranberries used for juice concentrates (i.e., normal to low fruit quality) have decreased. Of secondary importance were traits related to disease resistance (i.e., fruit rot), abiotic plant stresses, and insects. Interestingly, yield and productivity were not assigned as important priority, nor were fruit quality traits related to shelf-life, flavor, and sweetness (Gallardo et al., 2018). The development of a “sweet” cranberry could be the DG and the genetic change the cranberry industry needs to propel the emergence of a new cultural change via the creation of a cranberry fresh fruit market and consumer demand.

As this extended consideration of cranberry shows, integration of a *de novo* domesticate into a culture’s cuisine and food rituals and norms may be needed to continue the domestication process and sustain research investments in terms of agronomic and/or genetic change. While the exact size or appearance of newly domesticated food crops is difficult to predict prior to domestication, investigations into flavor, digestibility, toxicity and nutritional profile can help identify candidates or reveal additional biochemical pathways that need to be targeted for genetic change. Genetic and agronomic change remains central to work on intermediate wheatgrass, as described earlier. But a small amount of cultural change was seeded early on when the Rodale Institute began testing the nutritional and cooking properties of this new grain (Becker et al., 1991) and marketed it as “wild triga” (Wills et al., 1998). Likewise, The Land Institute and collaborators also conducted food science research (Marti et al., 2016; Banjade et al., 2019; Tyl et al., 2020). In line with previous efforts that led to the name wild triga, current marketers have expressed the need for a brief and memorable name. The Kernza trade name was initiated for this purpose, and Kernza branded grain has now been used in several restaurants, food products and beers. Customer demand for Kernza grain-containing products could, in the future, motivate corporate and governmental investment in further genetic and agronomic components of domestication.

### Value of Recognizing Cultural Change Involved in Domestication

As with agronomic change, attention to cultural change invites essential research and engagement with the humans who will grow, eat, trade, and continue to develop new crops. Without social engagement and support, appropriate tools and agronomic practices, or broadly adapted varieties, new crops will remain local and/or niche crops. Without a loyal constituency advocating for sustained research investment, they could easily be abandoned when new diseases appear or as the climate or the political and economic landscape changes. To produce the food security and agroecological benefits that new crops could provide, new crop acreage must eventually be substantial.

In order to enable ongoing social and economic support and later adoption, researchers should identify *de novo* domestication candidates that have the best chance of producing a positive return on the investments needed to domesticate and market them. Cultural and culinary uses of potential candidate plants may be documented in ethnobotany (Schlautman et al., 2018). From ethical, legal, and political standpoints as well as from a practical perspective, human relationships with candidate plants are relevant considerations in decisions about plant species’ suitability for domestication. Acknowledging the reality of cultural change affirms the ongoing importance of *in situ* conservation of landraces and of wild and weedy species on farms *with farmers* for new crop domestication, as human social factors are components of these complex systems (Casas et al., 2007; Bellon et al., 2017).

Recognition of established cultural components relevant to domestication can help identify promising opportunities for new crops. In Palestine, site of some of the earliest plant and animal domestication, wild plant gathering has continued to support human communities for millennia (Ali-Shtayeh et al., 2008; Eghbarieh, 2017; Research Collaborative, 2018). Wild food plants (Table 2) are important for traditional Palestinian cuisine and for sustenance during climatic and political crises (Tesdell et al., 2019). Palestinian people already value these plants in social, economic, and culinary terms (Tukan et al., 1998; Marouf et al., 2015). Recognition that these relevant cultural drivers for domestication may be in place is leading local groups to begin to collect seeds of edible wild plants and to explore ways to encourage more widespread cultivation beyond recent existing cultivation and transplanting practices (Doolittle and Mabry, 2006).

**TABLE 2:** Uncultivated plants commonly used in Palestinian cuisine.

**Vernacular name(s)**	**Scientific name**	**Family**	**Food type**
za’tar balat, duqah adas	*Clinopodium serpyllifolium (M. Bieb.) Kuntze*	Lamiaceae	Tisane, seasoning
za’atar	*Origanum syriacum* L.	Lamiaceae	Herb, seasoning, tisane
za’tar rumi, za’tar beid, za’itman	*Satureja thymbra* L.	Lamiaceae	Herb, seasoning, tisane
za’tar farisi	*Thymbra capitata* (L.) *Cav.*	Lamiaceae	Herb, seasoning, tisane
za’tar sabbal, sabbaleh	*Thymbra spicata* L.	Lamiaceae	Herb, seasoning, tisane
maramiyyeh	*Salvia fruticosa Mill.*	Lamiaceae	Herb, seasoning, tisane
Waraq lisan, lisseneh	*Salvia hierosolymitana Boiss.*	Lamiaceae	Cooking green
humeymsa	*Rumex cyprius Murb.*	Polygonaceae	Salad green
za’rur	*Crataegus azarolus* L.	Rosaceae	Fruit
qayqab	*Arbutus andrachne* L.	Ericaceae	Fruit
seyba’a, sneyba’a	*Lotus palaestinus Blatt.*	Fabaceae	Fresh vegetable, pulse
Burreid	*Pisum fulvum Sibth. & Sm.*	Fabaceae	Fresh vegetable, pulse
Khobs al ra’i, qurus sitti	*Medicago orbicularis* (L.) *Bartal*	Fabaceae	Fresh vegetable
kharrub	*Ceratonia siliqua* L.	Fabaceae	Sweetener
‘akkoub	*Gundelia tournefortii* L.	Asteraceae	Cooking vegetable
helyoun	*Asparagus aphyllus* L.	Asparagaceae	Cooking vegetable
za’matot, qarn al ghazal	*Cyclamen persicum Mill.*	Primulaceae	Cooking vegetable

*Tesdell, O. (editor). 2018 Palestinian Wild Food Plants.*

Several wild food plants could be used as grain legumes or oilseed grains, in addition to their current use as vegetables (Tesdell et al., 2020). Examples include *Lotus palaestinus* Blatt., *Pisum fulvum* Sibth. & Sm., and *Gundelia tournefortii* L. (‘akkoub, hereafter). ‘Akkoub, a wildcrafted and increasingly cultivated plant, offers opportunities as a perennial vegetable and possible oilseed among other uses. In addition, many crops remain widely foraged in Palestine and several surrounding countries. Table 2 offers a few prominent examples of Palestinian foraged plants of a list that could include more than 200 documented wild food plants to date. Their Arabic local names vary by region, but these wild plants persist as highly culturally important foods. Existing communities’ cultural valuation of this candidate plant could help enable and support pursuit of agronomic and genetic changes involved in *de novo* domestication.

In other *de novo* domestication efforts, it may be necessary to catalyze cultural change. Public interest, motivation, and support for *de novo* domestication may not be gained through simple, one-time, or purely rational means. Instead, building and sustaining the cultural change needed to support new crops may involve navigating a complex web of ongoing processes that include the affective, educational, ethical, legal, narrative, political, social, and other dimensions of human experiences and structures. Like scientific legitimacy more generally, agroecological legitimacy may be accomplished by meeting “credibility tests” and wisely engaging in this complex web of social life (De Wit and Iles, 2016). Honest acknowledgment and critical examination of value influences across the scientific research and development process, from inquiry to application, will likely be necessary for successful domestication of new crops – especially via gene editing (Elliott, 2017).

Participatory research and citizen science methods (Mendez et al., 2013; Ryan et al., 2018) – such as the involvement of many, geographically distributed farmers at an early stage of domestication – could help build initial communities that inform broader cultural support for the plant species. Introducing a potential food crop as a forage or specialty crop first may have the advantage of greatly increasing the number of farmers and other social groups involved and, at the same time, increasing the number of agricultural environments in which the species can be evaluated and selected.

One example of public support for new crop valuation is the Forever Green Initiative, developed at the University of Minnesota based on the principle that universities must engage with multiple stakeholders to realize their mission. When multiple stakeholders are considered, the broader societal impacts of agriculture must be considered. When agriculture threatens wildlife habitat or the provision of clean water or becomes a source of greenhouse gases, a broad base of stakeholders has the opportunity to support research directed at finding solutions to these challenges. The Forever Green Initiative has undertaken new domestication programs that aim to develop crops that will enable agricultural production to provide broad public goods. These efforts are gaining widespread interest and support (Runck et al., 2013; Anderson, 2014; Kleinhuizen, 2017; Haspel, 2019).

Documenting the ability of domestication candidates to provide regulating, cultural, and supporting ecosystem services (such as maintenance of soil and water quality, wildlife habitat, pollinator resources) could help build the case for social and economic valuation of new crops, including governmental support for continued research, farm payments, or the ability to charge consumers premium prices (Leakey and Asaah, 2013; Runck et al., 2014; Smýkal et al., 2018). As Kernza perennial grain has entered the market through the introduction of a few specialty products, abundant opportunities have emerged for storytelling around the new grain and its benefits. Messages about the ecological benefits of the grain have been placed directly on packaging such as beer cans and cereal boxes. Niche products have created a crowd funding project (Pierce, 2019) and generated numerous news reports (Bland, 2016; Garfield, 2016; Ostrander, 2017). Although this grain needs much additional research to enable profitable production at larger scale, niche specialty marketing boosts awareness and support, potentially inducing sustained funding.

In the case of *de novo* domestication that utilizes gene editing, special consideration should be given at an early stage to barriers to cultural adoption. Technological improvements in traditional crops have sometimes failed for cultural reasons. The reaction against transgenic foods leading to their non-adoption in Europe and elsewhere is an excellent example of failure to achieve cultural modification in support of a genetic approach (Kloppenburg, 2010). Social and educational researchers should investigate public perception and knowledge of the potential future domesticates via gene editing and consider strategies to foster informed public engagement in social decisions related to new crop domestication (Østerberg et al., 2017).

## Integrating Drivers of Change in Domestication

In contrast to the idea that gene editing can nearly instantly and/or inexpensively produce domestication, the established literature and our examples of intermediate wheatgrass, ‘akkoub, and cranberry demonstrate the multiple components involved in long-term domestication. Genetic, agronomic, and cultural changes interact over time to drive domestication processes. To illustrate lessons learned about how these changes are entangled in complex ways, we turn to a final personal narrative example of a current *de novo* domestication process. This narrative is not intended to provide recommendations for other new crop candidates, as each will enter the domestication “pipeline” (DeHaan et al., 2016) at a different stage and with different liabilities.

The Land Institute (Salina, KS, United States) has been domesticating *Silphium integrifolium* Michx. (silphium, hereafter) as a perennial oilseed crop since the early 2000s (Van Tassel et al., 2017). Human cultural valuation and ecological knowledge informed the decision to begin domestication, as local silphium populations were observed to have large, good-tasting seeds and to perform well during seasonal or year-long droughts. The latter observation was confirmed anecdotally by botanists in Texas (James Manhart, personal communication) and during the droughts of the 1930s by ecologist John Weaver (Weaver et al., 1935). Seeds from several wild populations in central Kansas were collected and grown in observation plots in Salina and allowed to intermate to produce seeds. The experimental plants grew much larger than plants in nearby prairies, suggesting that silphium is well-adapted to agricultural conditions and amenable to agronomic change.

However, domestication efforts first focused on driving genetic change. The disk florets of silphium are staminate, limiting the number of seeds per head to 15–25 (equal to the number of ray florets). Therefore, the first breeding target in 2004 was to generate and identify a mutation causing bisexual disk florets or to use recurrent selection to increase the number of ray florets (Van Tassel et al., 2014). Mutagenesis was attempted but no mutants with the phenotype of interest were recovered. However, several cycles of recurrent visual selection on ligule number (ray floret number) were successful in feminizing the heads of silphium to a point where some individuals were almost male-sterile. In 2012, parents for new cycles of selection were made for partial feminization plus increased achene (hereafter “seed”) size and head diameter. Recurrent selection for non-dormant seed was attempted and reduced shattering was a breeding goal. In short, traits comprising the classic domestication syndrome were targeted, albeit mostly with the expectation of finding highly polygenic control of these traits as reported in sunflower (Burke et al., 2002). These included a greater number of larger heads and seeds, non-dormant seeds, and higher seed oil content.

In 2014 and every year thereafter, plants in research plots in Salina Kansas were infected with rust (*Puccinia silphii*) and other pathogens that have yet to be conclusively identified. *Eucosma giganteana*, a specialist moth whose larvae are head and crown parasites of several *Silphium* species, also appeared and became a serious pest with colonization of heads approaching 100% in many situations. These pests and pathogens may be more common in more humid environments and did not become common in Salina until we had created favorable microclimates (lush, dense stands) and large populations of host plants (Turner et al., 2018; Vilela et al., 2018).

The pest and pathogen pressures have made selection for yield or domestication traits difficult in Salina. In genetic terms, improved strains and breeding populations at The Land Institute have little variation for resistance to several pathogens and pests and although these populations indeed have higher seed yield than wild silphium, increased yield is accompanied by increased plant size. Standard harvesting machinery does not work as well with taller plants with stouter stalks, and increased biomass almost certainly implies greater water use. In agronomic terms, attempts to scale up plot sizes by direct seeding have had highly variable success and even transplanting in new fields has occasionally failed for unknown reasons. Together these limitations have made it hard to obtain quantities of seed needed for food or feed science research – i.e., as a strategy to drive cultural valuation.

Renewed collection of wild silphium from throughout its natural range has revealed individual plants or populations that appear to have greater resistance to a number of pests or pathogens; some also appear to be shorter and have more slender stems that may be more compatible with combine harvesters. However, these ecotypes from south or east of Kansas have much smaller seeds and smaller, less feminized seeds and heads.

Looking back, we see that the western (Kansas) ecotype attracted us (humans) with its unusually large heads and seeds. However, the initial decision to focus exclusively on genetic change for classic domestication traits, especially seed and head size, at this point distracted us from first diversifying the genetic base of the breeding populations (which would have meant reducing average seed and head size) or discovering specialized agronomic management practices as would have been necessary to first achieve broad adaptation or cultural adoption of silphium as a forage or other specialty crop.

In the United States, this kind of work has been done for many new crops, beginning with the USDA’s Section of Seed and Plant Introduction in 1898 and in partnership with germplasm “banks” and agricultural universities (Hyland, 1977). However, many native plants, including *Silphium integrifolium* have not received such attention and perhaps we should have recognized the need to perform this work ourselves and spent several years collecting more diverse germplasm, increasing it, and testing it in multiple environment to identify vigorous, resistant, and stable lines.

Another alternative scenario would have been to prioritize agronomic physiology and horticultural research over genetic selection during the initial stage of domestication. Although average seed yield in Kansas was about 300 kg/ha (prior to pest outbreaks), 1600 kg/ha was achieved in one year and one location in Minnesota (Schiffner, 2018). Clearly, the species already has the genetic potential to yield well. We might have been better served spending the time to understand what environmental conditions contribute to the “yield gap” between this potential and average yield than rushing ahead with selection for increased yield potential.

Some domesticated plant phenotypes can be produced through careful management of wild germplasm. For example, surgical manipulation of plant size and branching pattern is an ancient art still actively practiced with many woody tree and vine crops. We have had some promising results trimming of silphium stalks to reduce stalk height and improve architecture, but the procedure is very sensitive to pruning height and timing. This is an excellent example of how humans develop detailed, species-specific horticultural practices, formally or informally, much of it by trial-and-error.

Furthermore, there may be some advantages in retaining developmental plasticity for plant height and branching pattern, relying on skilled farmer management to produce harvestable, seed-productive phenotypes. This is because silphium has nutritious, palatable foliage and left unpruned could be used as a forage crop. Being able to use management to switch between forage and oilseed production could allow farmers to respond to weather or market fluctuations. Deliberately alternating between allowing heads to produce seeds (oilseed) and chopping the biomass prior to seed set (forage) may interrupt the lifecycle of specialist insects.

Finding another use for silphium prior to pursuing genetic changes necessary for domestication as a perennial oilseed crop could allow us to expand the number of people interacting with this species and the number of environments in which it could be tested. As an expanding ornamental, pollinator crop, or forage crop, seed production practices would still need to be developed to make seed more available and affordable. This demand could stimulate agronomic innovation in crop management and agricultural engineering and begin the process of building traditions, networks, companies and other social structures needed for the long-term success of a new crop.

These lessons learned about the public interest and support that will be needed to sustain agronomic and genetic change in silphium have encouraged us to experiment with methods to investigate and initiate cultural change. Citizen science has been identified as a way to meet “grand challenges” in agriculture (Ryan et al., 2018). Providing people with access to potential new crops-in-process could help build interest and increase knowledge and awareness. Participants could become a publicly visible and informed network of collaborators. Learning outcomes gained through long-term citizen science projects related to new crop domestication could catalyze and fuel the cultural change needed to enable and sustain domestication.

We began a silphium civic science pilot community in 2019. More than 40 people in 18 states joined. Like farmers who participate in on-farm research, participants in our silphium civic science community grow seedlings in backyards, gardens, farms, and public spaces and collect data over multiple seasons in a range of environments. They also share and reflect on their own personal experiences, interests, and questions about silphium’s relationship with insects, soils, mammals, and humans – including its future uses by humans. Participants receive educational materials and can interact and learn with plants, with each other, and with scientists and educational researchers. While further research is needed, preliminary results indicate positive engagement.

Silphium is not currently a good candidate for gene editing because its large, intractable genome has limited genetic and genomic research in the species, because obvious monogenic DGs have not been identified in its closest crop relative, *H. annus* (Dowell et al., 2019), and because pests, diseases and soil fertility prevent silphium from achieving its existing seed yield potential in field conditions. Rather, silphium reaffirms the persistent need for science and society alike to understand the complexity of new food crop domestication. Successful *de novo* domestications will likely be the result of diverse, adaptive approaches to integrating genetic, agronomic, and cultural drivers of change over time.

## Conclusion

In the preceding sections we have highlighted how *de novo* crop domestication, ranging from historical to contemporary, can be viewed as the varied and ongoing interaction of three factors of innovation: genetic, agronomic, and cultural. Gene editing approaches to domestication using DGs risk portraying a misunderstanding of *de novo* crop domestication as something humans do to wild plants in a simple one-time event, in a single gene or generation, using increasingly cheap technology, and drawing from just a few scientific disciplines. In contrast, *de novo* domestication literature and examples (Table 3) understand domestication as co-evolutionary interactions between plants and peoples that are complex, stretch across generations indefinitely, may require significant institutional and infrastructural investments, and can involve many disciplines and ways of knowing. This knowledge contextualizes gene editing approaches by remembering the additional work and integrated knowledge needed to accomplish goals that are shared across research communities: the accelerated domestication and widespread cultivation of a new generation of soil-conserving and climate-smart crops.

**TABLE 3:** Summary of examples of genetic, agronomic, and cultural change for plant domestication projects discussed in this article.

**Example**	**Genetic**	**Agronomic**	**Cultural**
‘Akkoub (*Gundelia tournefortii*)	Very few accessions have been collected by gene banks	A few gardeners are beginning to cultivate it	Enthusiastically harvested from the wild for home use and for sale as a vegetable and medicinal; at risk of overharvesting and habitat loss; access to habitat increasingly restricted
Cranberry (*Vaccinium macrocarpum*)	Superior wild genotypes are still being clonally propagated; breeding programs are relatively new	Farmers have been constructing and irrigating bogs for about 100 years; advances in management and harvesting techniques increased yield and reduced costs	Indigenous peoples have long harvested fruit from wild bogs and have cultural knowledge about its culinary and medicinal value; colonists appropriated this knowledge and developed additional uses and markets
Intermediate wheatgrass (*Thinopyrum intermedium*)	Introduced as a forage and improved varieties released since the 1940s; recurrent selection for use as a cereal grain is ongoing	Agronomic research resulted in recommendations for forage management; grain production research is ongoing	Few people know this species but new breakfast cereal and beer products are being marketed under the Kernza name to increase awareness
Silphium (*Silphium integrifolium*)	Recurrent selection for seeds per head and other agronomic traits for the past 20 years	Not cultivated until breeding program began; a few small agronomic studies have been published	Indigenous knowledge and medicinal and cultural use, but no documented use as a food; civic science pilot project recently initiated
Currant tomato (*Solanum pimpinellifolium*)	CRISPR/Cas9 editing of 6 genes		

In the age of gene editing and at this moment of decision-making about domestication pathways, new food crop domestication might be viewed as analogous to a new public health campaign, in all its complexity. Narratives calling for new vaccines are exciting and easily communicated to private and public investors. But advances in vaccine molecules must be accompanied by sustained monitoring of virus evolution and transmission, development of improved vaccine delivery technology, support for clinics and vaccination campaigns and cultural work toward acceptance and use of vaccinations. Investment in novel recombinant or DNA vaccines could accelerate vaccine development or enable vaccination for new diseases, but this investment will not be worthwhile if vaccines are rejected on cultural or religious grounds, if vaccines are not reformulated to reflect pathogen evolution, or if vaccines can’t be efficiently delivered to vulnerable populations. Similarly, recognizing the continuing importance of interacting genetic, agronomic, and cultural drivers to successful *de novo* domestication, we call on funding agencies, proposal reviewers and authors, and research communities (of new crop domesticators, growers, and consumers) to value and support agroecology, agronomy, plant breeding, and participatory, interdisciplinary, and transdisciplinary approaches alongside genetics and genomics.

## Author Contributions

DV, AS, BS, OT, MR, LD, and TC: framework conceptualization, research, writing, and editing. DV: silphium and intermediate wheatgrass case studies. AS: silphium case study. BS: cranberry case study. OT: Palestinian wild foods case study. LD: intermediate wheatgrass case study.

## Conflict of Interest

The authors declare that the research was conducted in the absence of any commercial or financial relationships that could be construed as a potential conflict of interest.
